# Risk prediction for dermatomyositis-associated hepatocellular carcinoma

**DOI:** 10.1186/s12859-023-05353-6

**Published:** 2023-05-31

**Authors:** Xusheng Zhang, Yongxin Ma, Kejun Liu, Long Chen, Lin Ding, Weihu Ma, Bendong Chen

**Affiliations:** 1 Ningxia Medical University, Yinchuan, 750004 China; 2 Department of Hepatobiliary Surgery, General Hospital of Ningxia Medical University, Yinchuan, 750004 China; 3 Ningxia Hepatobiliary and Pancreatic Surgical Diseases Clinical Medical Research Center, Yinchuan, 750004 China

**Keywords:** Hepatocellular carcinoma, Dermatomyositis, PI3K/ART signaling pathway, SPP1, Support vector machine

## Abstract

**Objective:**

To explore dermatomyositis signature genes as potential biomarkers of hepatocellular carcinoma and their associated molecular regulatory mechanisms.

**Methods:**

Based on the mRNA-Seq data of dermatomyositis and hepatocellular carcinoma in public databases, five dermatomyositis signature genes were screened by LASSO regression analysis and support vector machine (SVM) algorithm, and their biological functions in dermatomyositis with hepatocellular carcinoma were investigated, and a nomogram risk prediction model for hepatocellular carcinoma was constructed and its predictive efficiency was initially evaluated. The immune profile in hepatocellular carcinoma was examined based on the CIBERSORT and ssGSEA algorithms, and the correlation between five dermatomyositis signature genes and tumor immune cell infiltration and immune checkpoints in hepatocellular carcinoma was investigated.

**Results:**

The expression levels of five dermatomyositis signature genes were significantly altered in hepatocellular carcinoma and showed good diagnostic efficacy for hepatocellular carcinoma, suggesting that they may be potential predictive targets for hepatocellular carcinoma, and the risk prediction model based on five dermatomyositis signature genes showed good risk prediction efficacy for hepatocellular carcinoma and has good potential for clinical application. In addition, we also found that the upregulation of SPP1 expression may activate the PI3K/ART signaling pathway through integrin-mediated activation, which in turn regulates the development and progression of hepatocellular carcinoma.

**Conclusion:**

LY6E, IFITM1, GADD45A, MT1M, and SPP1 are potential predictive targets for new-onset hepatocellular carcinoma in patients with dermatomyositis, and the upregulation of SPP1 expression may activate the PI3K/ART signaling pathway through the mediation of integrins to promote the development and progression of hepatocellular carcinoma.

**Supplementary Information:**

The online version contains supplementary material available at 10.1186/s12859-023-05353-6.

## Introduction

Dermatomyositis (DM) is a type of idiopathic inflammatory myopathy (IIM), which is characterized by a multisystem involvement of the skin and muscles and can occur in both adults and children [1], and DM with malignant neoplasm is more common in clinical practice and is one of the main subtypes of DM [2]. Studies have shown that patients with DM have a significantly higher risk of developing malignant tumors compared to the healthy population, with approximately 15–30% of DM patients suffering from associated malignancies [3], and the pathogenesis of DM with malignant tumors has not been investigated to date, with some studies hypothesizing that it may be related to autoimmune disorders, TIF1 gene mutations and heterozygous deletions, and reduced immune surveillance capacity of the body [4, 5].

Hepatocellular carcinoma (HCC) is the most common primary liver cancer, accounting for approximately 75–85% of it [6]. According to the latest statistics, HCC ranks sixth among all cancers and is also the third most lethal cancer, while domestic statistics show that primary liver cancer ranks as the fourth most common malignant tumor and the second most lethal cause of tumor, seriously threatening human life and health [7, 8].

In the course of clinical work, we found that patients with DM seem to be more inclined to develop HCC, and through a literature search we found that cases of DM with HCC are often reported [9–11], but similar to other tumors, the pathogenesis of HCC in patients with DM has not been investigated so far. Based on this, our study attempted to investigate the association between the two at the genetic level to provide useful references for the diagnosis and treatment of DM patients with HCC and subsequent clinical studies.

## Materials and methods

### Study subjects

The gene expression profile data of HCC relevant to our study were obtained from the HCC mRNA-Seq data in the TCGA (https://portal.gdc.cancer.gov/) database and the GTEx (https://www.gtexportal.org/home/index.html) database of normal liver mRNA-Seq data, GEO (https://www.ncbi.nlm.nih.gov/geo/) database for dermatomyositis dataset GSE46239 with GSE1551 (Additional file 1), and HCC dataset GSE14520 with GSE36376 mRNA-Seq (Additional file 2). The data of TCGA, GTEx, and GEO databases are publicly available. Therefore, no local ethics committee approval was required for this study. The present study follows the official TCGA, GTEx, and GEO data access policy and publication guidelines.

### R-based analysis

The R “limma” package was used to analyze the differentially expressed genes in DM and HCC and to draw heat maps and volcano maps. SVM algorithm and LASSO regression analysis of differentially expressed genes in both diseases were performed using the R “e1071” package and “glmnet package” respectively, diagnostic efficacy of genes was analyzed using R “pROC” package and diagnostic ROC curves were drawn, R “rms” and “rmda” packages were used to construct risk nomogram models were constructed and validated.

### Human protein atlas (HPA)-based analysis

The Human Protein Atlas (HPA) (https://www.proteinatlas.org/) contains antibody-based imaging, mass spectrometry for proteomics, transcriptomics, and systems biology, and all data in the knowledge resource are open access. We used this database to further explore the expression of DM signature genes in HCC.

### Statistical analysis

Statistical analyses were performed using R, and plots were drawn using Adobe Illustrator 2020. The measurement data conforming to normal distribution were expressed as mean ± standard deviation, and the Wilcoxon rank sum test was used for comparison between groups. The Chi-square test was used for comparison between groups of count data, paired samples were analyzed using paired t-test. Spearman correlation analysis was used for simple correlation analysis of measurement data not conforming to a normal distribution, Fisher exact test was used for data of small sample groups, and the rank sum test was used to compare groups of measurement data that did not conform to a normal distribution. *P* < 0.05 was statistically significant.

## Results

### Analysis of differentially expressed genes in DM and HCC

**Fig. 1:**
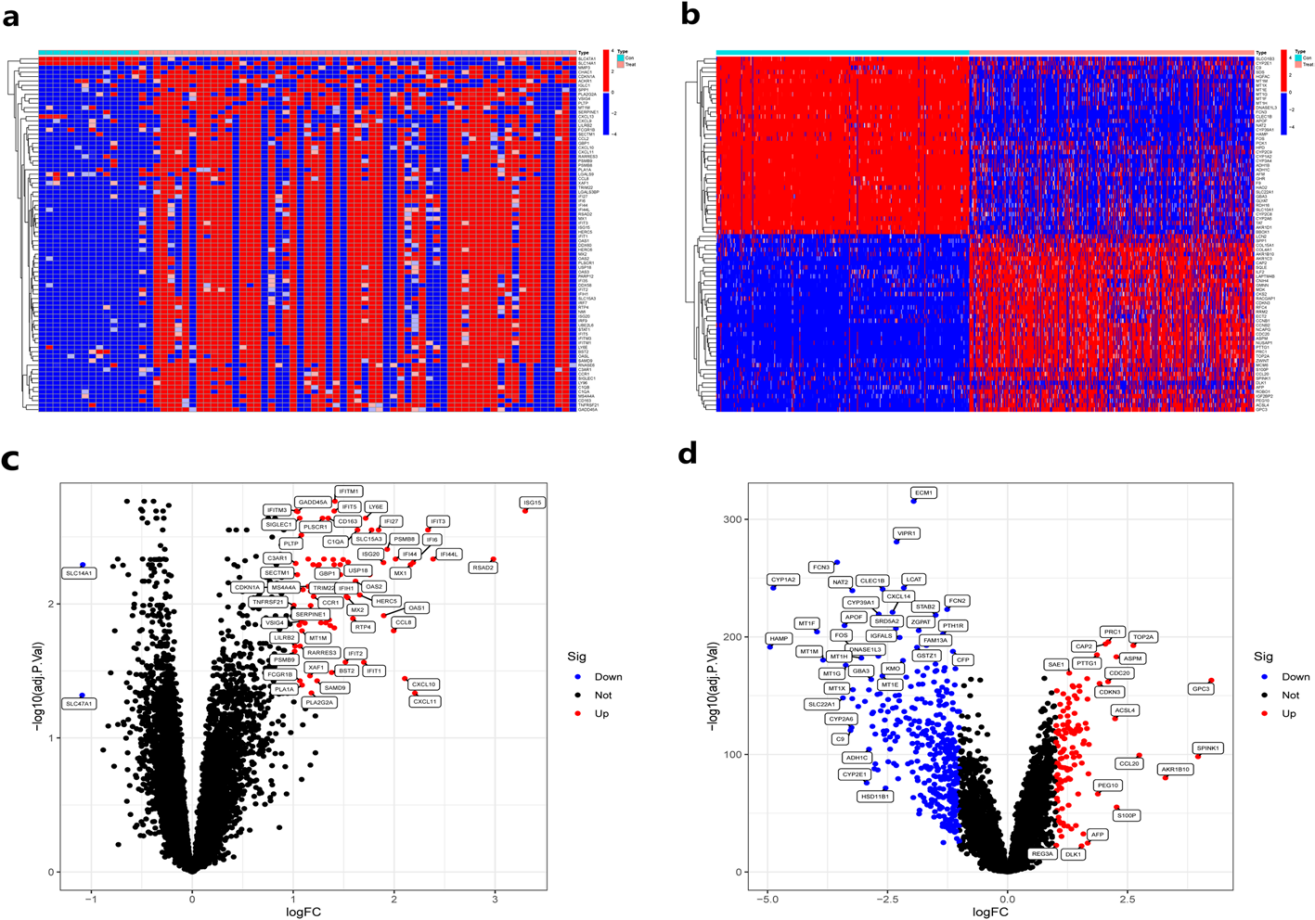
Analysis of differentially expressed genes in DM and hepatocellular carcinoma. **a**, **b** Heat map of differentially expressed genes. **a** DM, **b** HCC. **c, d** Volcano map of differentially expressed genes. **c** DM, **d** HCC

The mRNA-Seq datasets GSE46239 and GSE1551 for DM, and GSE14520 and GSE36376 for HCC were downloaded from the GEO database. The data of DM and HCC were sorted and normalized respectively, and then the differential expression analysis was performed on the mRNA expression data of the two diseases with the setting condition of LogFC ≥ 1, *P* < 0.05, the results showed that there were 80 differentially expressed genes in DM(Additional file 3) and 480 differentially expressed genes in HCC (Additional file 4), and then heat maps (Fig. 1a, b) and volcano maps (Fig. 1c, d) were plotted for the differential gene expression in two diseases, respectively. Following that, we further explored the common differentially expressed genes in DM and HCC and took the intersection of differentially expressed genes in both, and the results showed that six genes were differentially expressed in both diseases (Additional file 5: Fig. S1).

### Characteristic gene screening

**Fig. 2:**
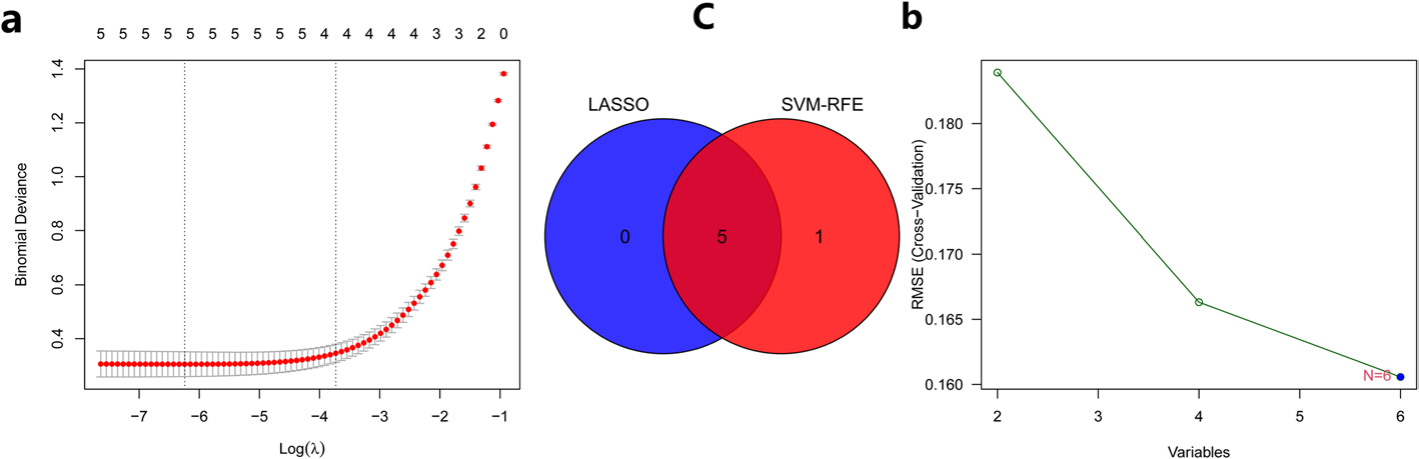
Characteristic gene screening. **a** Results of LASSO regression analysis, and 5 genes were obtained. **b** Results of SVM analysis, and 6 genes were obtained. **c** Venn diagram of the intersection of the results of the two algorithms, and 5 genes were obtained. Con:Normal tissure. Treat: Disease

After the initial screening, we found six genes in common among the differentially expressed genes of DM and HCC, and then we used LASSO regression analysis and support vector machine (SVM) machine learning algorithm to screen the six intersecting genes based on the expression of the six intersecting genes, and the results showed that five genes were obtained after LASSO regression analysis (Fig. 2a), and six genes were obtained after SVM calculation (Fig. 2b). We then took the intersection of the results of both algorithms again, and the results showed that five genes were screened in both algorithms (Fig. 2c), which were IFITM1, GADD45A, LY6E, TRIM22, MT1M, and SPP1, and these five genes were identified as common characteristic genes of DM and HCC.

### Characterized gene expression analysis in DM and HCC

**Fig. 3:**
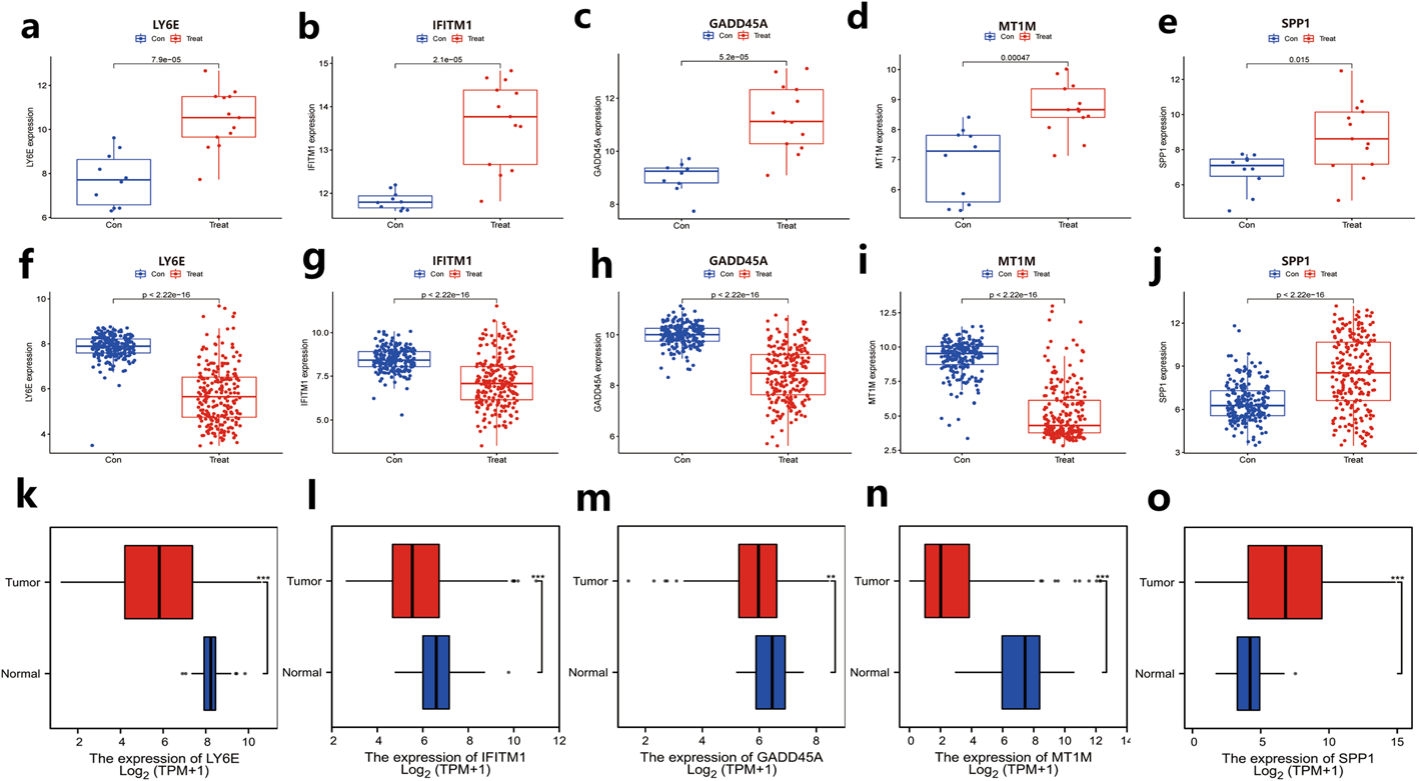
Expression analysis of signature genes in DM and HCC. **a–e** Expression analysis of five signature genes in DM, a-e in order are LY6E, IFITM1, GADD45A, MT1M, and SPP1. **f–j** Expression analysis of five signature genes in HCC, in order are LY6E, IFITM1, GADD45A, MT1M, and SPP1. **k–o** Expression analysis of 5 signature genes in HCC validated by TCGA data, in order LY6E, IFITM1, GADD45A, MT1M, and SPP1. **P < 0.005, ***P < 0.001. Con: Normal tissure. Treat: Disease (DC or HCC)

After identifying the common characteristic genes of DM and HCC, we further analyzed the expression of IFITM1, GADD45A, LY6E, MT1M, and SPP1 in both diseases. The results showed that the expression levels of IFITM1, GADD45A, LY6E, MT1M, and SPP1 were significantly upregulated in DM compared to the normal population (Fig. 3a–e, *P* < 0.05), whereas in HCC, the expression levels of IFITM1, GADD45A, LY6E, and MT1M were significantly downregulated except for SPP1 (Fig. 3f–j, *P* < 0.005). To verify the authenticity of this result, the expression of the five characteristic genes was further verified by using the HCC mRNA-Seq data from the TCGA database, and the same conclusion was finally obtained (Fig. 3k–o, *P* < 0.005).

### Analysis of diagnostic efficacy of signature genes in HCC

**Fig. 4:**
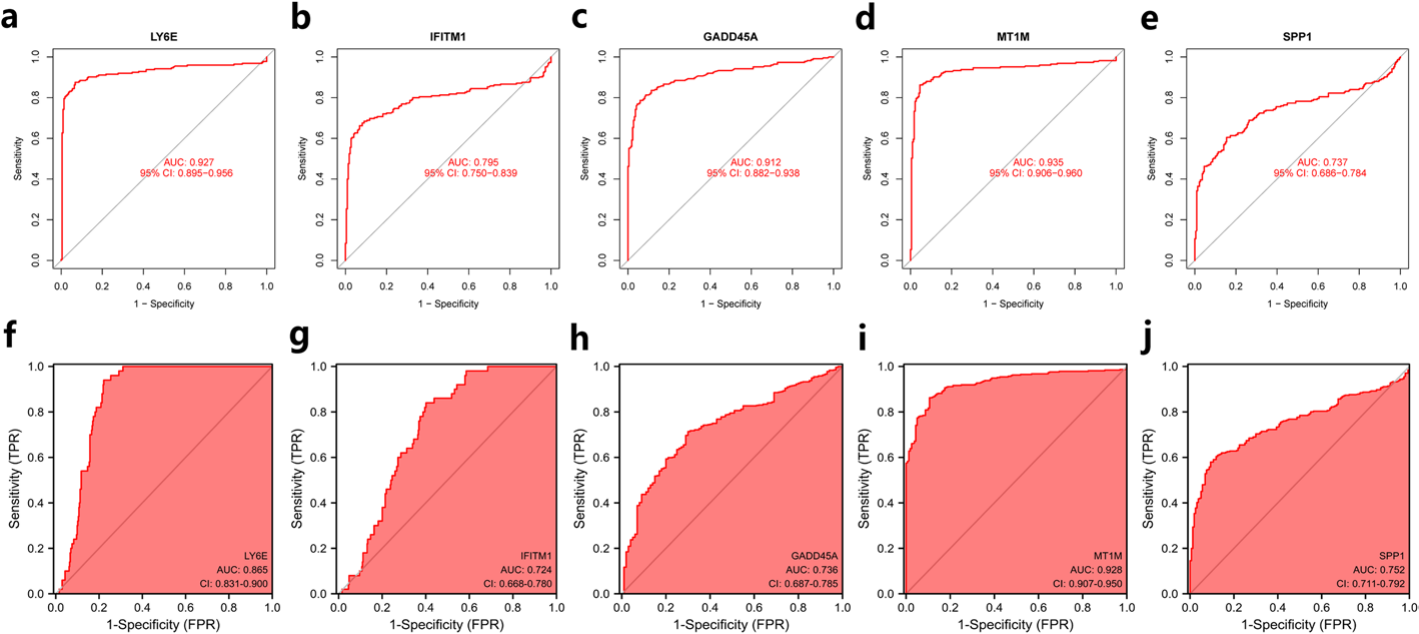
Diagnostic efficacy analysis of signature genes in HCC. **a–e** Diagnostic efficacy analysis of five signature genes in HCC of GEO dataset, in the order of LY6E, IFITM1, GADD45A, MT1M, and SPP1. **f–j** Diagnostic efficacy analysis of 5 signature genes in HCC of TCGA database, in the order of LY6E, IFITM1, GADD45A, MT1M and SPP1

After determining the expression of the signature genes, we further evaluated their diagnostic efficacy in HCC using GEO data, and the results showed that the diagnostic ROC-AUC of the five genes in HCC were all greater than 0.7, showing good diagnostic efficacy (Fig. 4a–e), and to verify the analysis results, we also analyzed the diagnostic efficacy of the five signature genes using TCGA database HCC data. The results showed that the diagnostic ROC-AUC was also over 0.7, which was consistent with the results of GEO data analysis (Fig. 4f–j), suggesting that LY6E, IFITM1, GADD45A, MT1M, and SPP1 are potential predictive targets for HCC.

### Construction and validation of the nomogram risk model

**Fig. 5:**
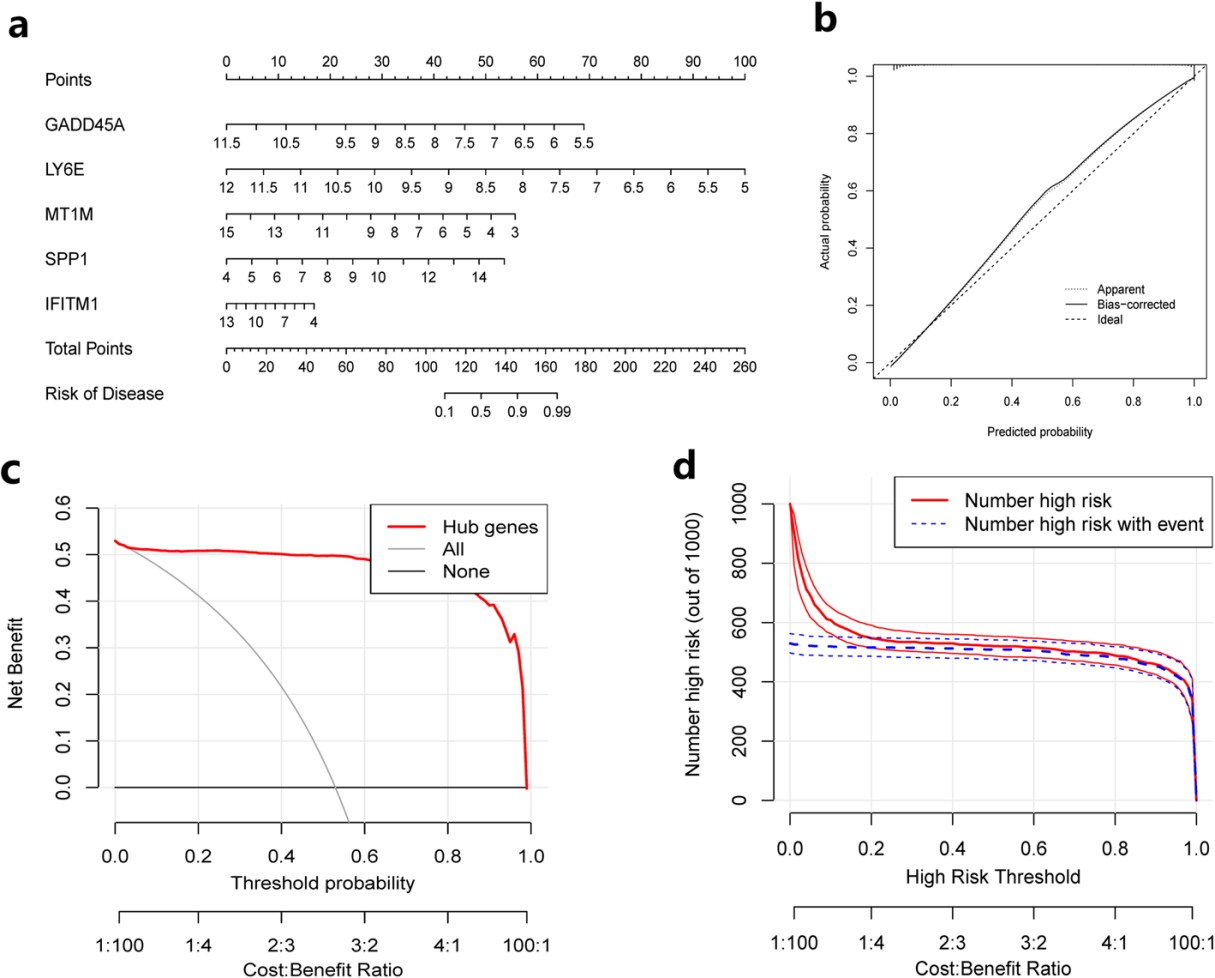
Construction and validation of the column line diagram risk model. **a** Nomogram of the risk prediction model. **b** Calibration curves. **c** Decision curve (DCA). **d** Clinical impact curves

After determining that LY6E, IFITM1, GADD45A, MT1M, and SPP1 all showed good diagnostic efficacy for HCC, the present study constructed a nomogram model for risk prediction of HCC based on the expression levels of the five characterized genes (Fig. 5a). As seen from the nomogram, the downregulated expression of LY6E, IFITM1, GADD45A, and MT1M and the upregulated expression of SPP1 were all risk factors for HCC and promote the risk of HCC. Following that, we performed a preliminary validation of the predictive efficacy of this line graph risk model. As shown in Fig. 5b, the calibration curves showed a good fitness suggesting a good predictive efficacy of the risk model, followed by a decision curve (DCA) to assess the net clinical benefit of the model, which showed a high net clinical benefit (Fig. 5c), and we also observed in the clinical impact curve that the true positive detection rate of the model was very close to the true positive rate, this suggests that the model has a high disease specificity (Fig. 5d), and collectively, the risk model shows good predictive performance and has a high potential for clinical application.

### HCC subtype analysis based on characteristic genes

**Fig. 6:**
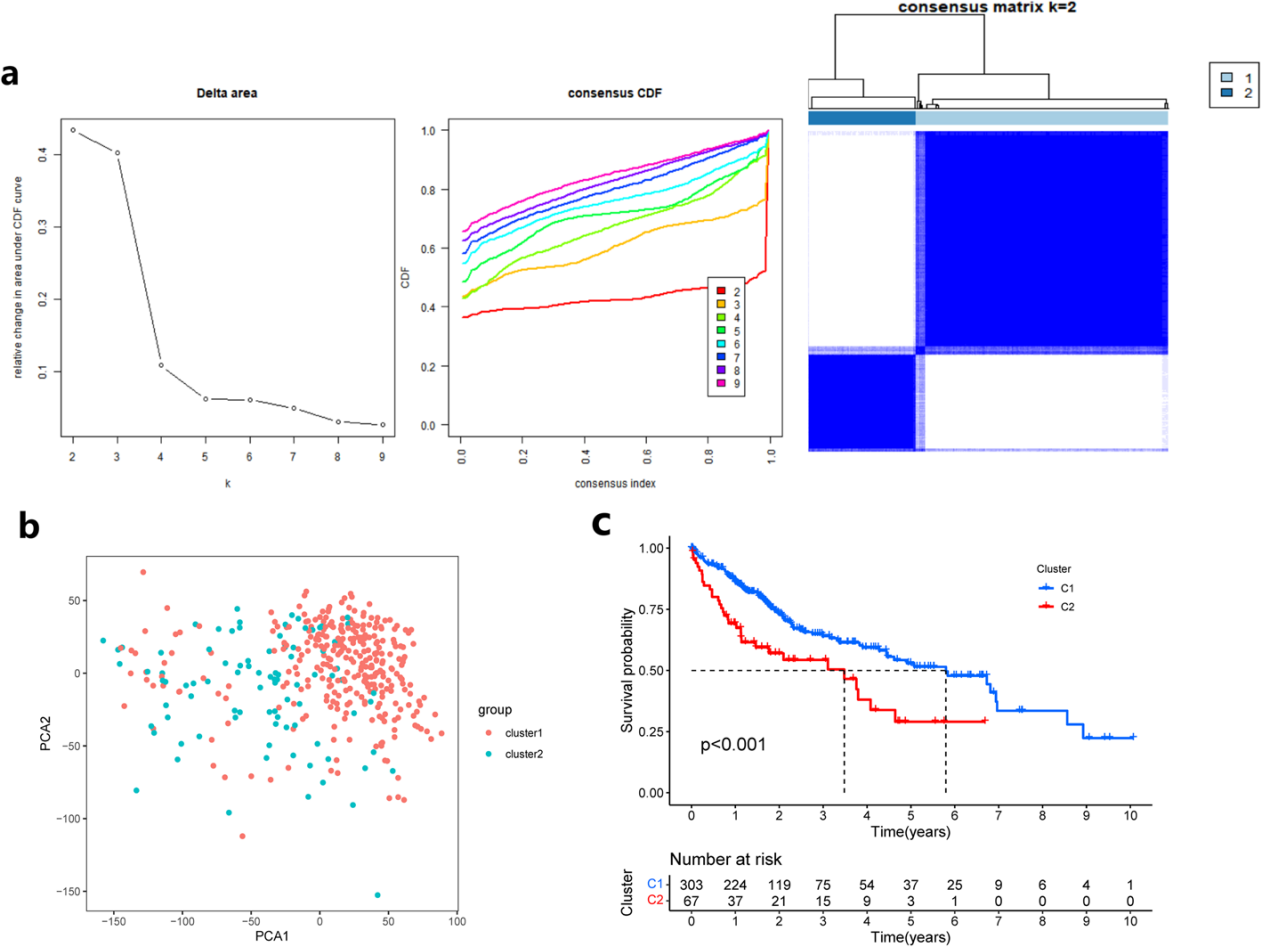
Subtype analysis of HCC based on characteristic genes. **a** Unsupervised cluster analysis. **b** Principal component analysis (PCA) scatter plot. **c** Survival analysis curve of two subtypes

Based on the mRNA expression levels of LY6E, IFITM1, GADD45A, MT1M, and SPP1 in HCC, we further performed subtype analysis of HCC using unsupervised clustering analysis, and the results classified all samples into Cluster 1 and Cluster 2 subtypes (Fig. 6a). To verify the reliability of the subtype classification results, we also did a principal component analysis (PCA) on all samples, and the results showed that all HCC samples were also clearly classified into two subgroups, Cluster 1 and Cluster 2 (Fig. 6b), which was consistent with the results of cluster analysis, suggesting that the expression levels of the five characteristic genes in HCC had a good typing effect on HCC. And our study also analyzed the survival between the two subtypes, and the results showed that there was a significant difference in the survival prognosis between the two subtypes, and Cluster 1 had a better survival prognosis (Fig. 6c).

### Immunological level and checkpoints of the two subtypes

**Fig. 7:**
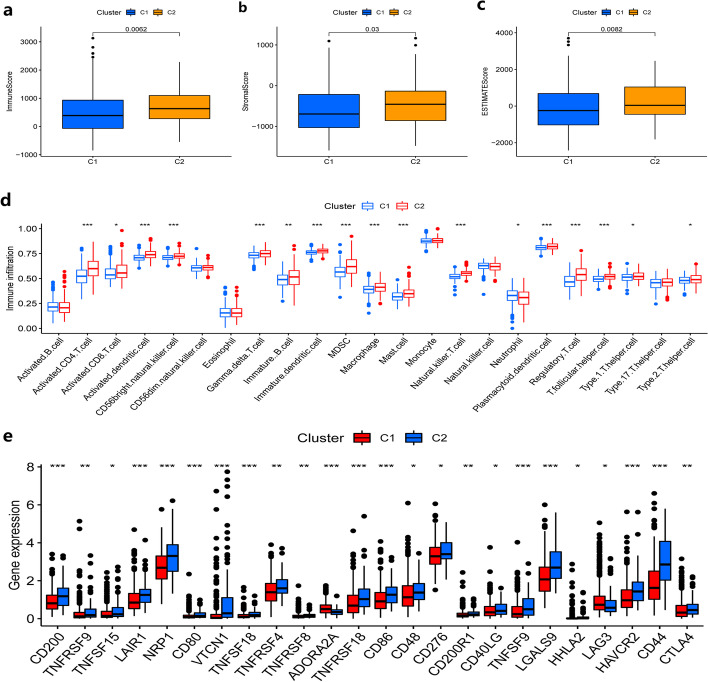
Immunological level analysis of the two subtypes and correlation analysis of immune checkpoints. **a–c** Boxplot plots of tumor microenvironment analysis for the two subtypes. **a** Tumor microenvironment immune score of two subtypes. **b** Tumor microenvironment stroma score of two subtypes. **c** Overall tumor microenvironment score of two subtypes. **d** Boxplot plot of immune cell infiltration level analysis between the two subtypes. **e** Boxplot plot of immune checkpoint expression level analysis between the two subtypes. *p < 0.05, **p < 0.005, ***p < 0.001

After subtyping the samples, we further explored the tumor immune profile between the two subtypes and analyzed the composition of the microenvironment, and the results showed that there were significant differences between the two subtypes C1 and C2 in both immune scores, stromal scores, and overall scores in the tumor microenvironment, and that C2 received higher scores in all three scoring groups (Fig. 7a–c). Further, we also analyzed the infiltration levels of tumor immune cells between the two subtypes based on the ssGSEA algorithm, and the results showed that the infiltration levels of more than ten types of immune cells, including activated B cells, activated CD 4 T cells, activated CD 8 T cells, activated DC cells, NK cells, macrophages, and mast cells, were significantly different between the two subtypes (Fig. 7d, *P* < 0.05). In addition, the relationships between the two subtypes and common immune checkpoints were also analyzed, and the results showed that the expression of dozens of immune checkpoints, including some classical immune targets such as CTLA4, differed between the two subtypes, and the C2 subtype appeared to be associated with higher expression of immune checkpoints (Fig. 7e, *P*  < 0.05).

### Analysis of immunization status of HCC

**Fig. 8:**
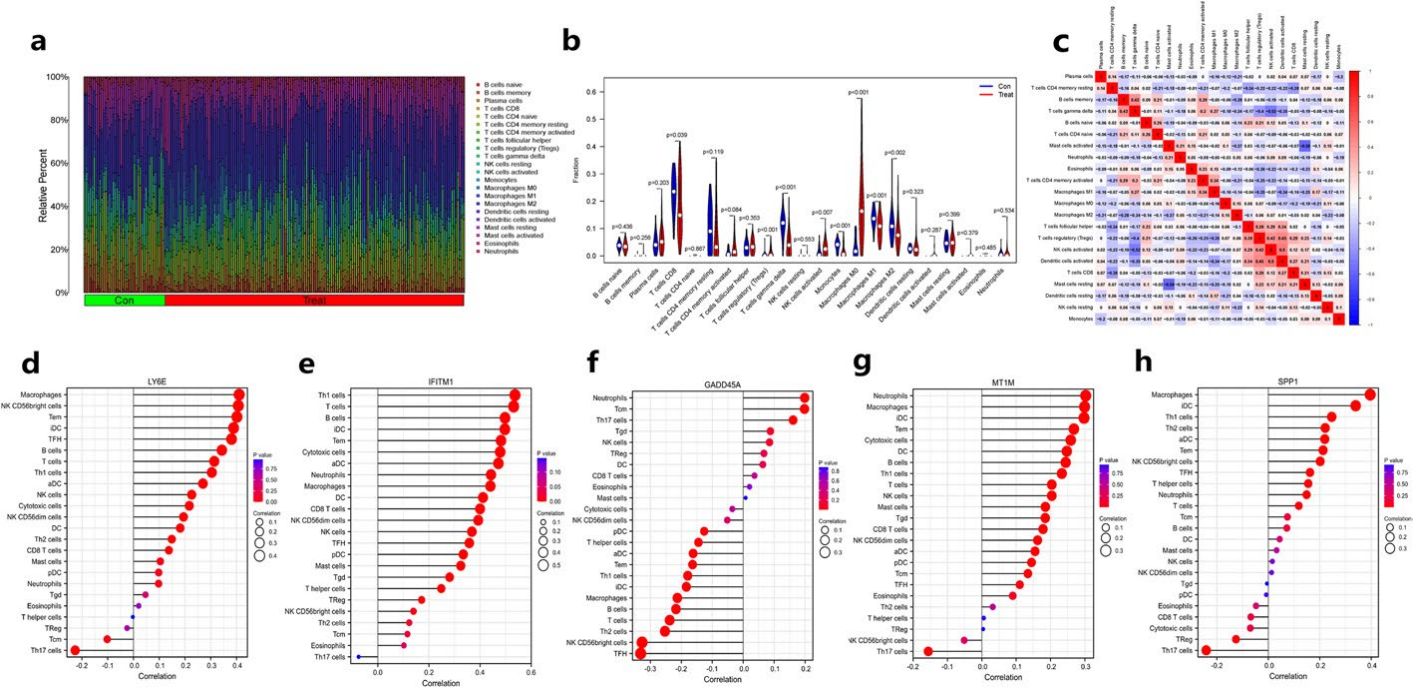
Immunological analysis of HCC. **a** Composition of immune cells in normal liver tissue versus HCC. **b** Violin plot of tumor immune cell infiltration in normal tissue compared to HCC. **c** Heat map of correlation analysis between each tumor immune cell in HCC. **d–h** Lollipop figure of immune infiltration analysis. Figure d-h in the order of LY6E, IFITM1, GADD45A, MT1M and SPP1. Con:Normal tissure. Treat: HCC

Tumor immunity is an important part of the tumor biological process, and the immune response in the tumor microenvironment has an important feedback role on the disease development and treatment of tumors, which is closely related to patient prognosis. In this research, we also analyzed the composition of immune cells in the tumor microenvironment of HCC based on CIBERSORT, as shown in Fig. 8a, the percentage of Macrophages M0, activated NK cells, Tregs, etc. were elevated in HCC compared with normal tissues. And further analyzed the difference of expression of each immune cell in normal tissues compared with HCC, the infiltration levels of Macrophages M0, activated NK cells, and Tregs were significantly increased, while the infiltration levels of CD 8 T cells, gamma-delta T cells, monocytes, Macrophages M1, and Macrophages M2 were significantly decreased (Fig. 8b, *P* < 0.05). Following this, we also analyzed the relationships between different immune cells, and the results showed that except for positive correlations between Tregs and activated NK cells, activated NK cells and activated DC cells, gamma-delta T cells and B memory cells (r > 0.4), other cells tended to have more negative correlations, but the correlation was generally not significant among immune cells (Fig. 8c, |r|< 0.4), which may be related to the overall tumor immune profile in HCC.

In addition, the correlation of five signature genes with the infiltration abundance of common tumor immune cells was analyzed based on TCGA HCC mRNA-seq data in present study, and the results showed that LY6E showed a significant positive correlation with immune cells such as Tem, NK CD56bright cells, Macrophages (r > 0.4, *P* < 0.001, Fig. 8d). IFITM1 showed a significant positive correlation with CD8 T cells, DCs, Terms, Macrophages, Neutrophils, aDCs, Cytotoxic cells, iDCs, B cells, T cells, and Th1 cells (r > 0.4, *P* < 0.001, Fig. 8e). And GADD45A showed a significant negative correlation with the abundance of immune cells infiltrated mainly with NK CD56 bright cells and TFHs (r < − 0.3, *P* < 0.001, Fig. 8f). MT1M showed a weak positive correlation with Cytotoxic cells, Tems, iDCs, Macrophages, and Neutrophils (r > 0.25, *P* < 0.001, Fig. 8g). And SPP1 showed a positive correlation mainly with iDCs and Macrophages (r > 0.3, *P* < 0.001, Fig. 8h). It should be noted that the expression levels of LY6E, IFITM1, GADD45A, and MT1M were downregulated in HCC, therefore, except for the expressions of GADD45A and SPP1, which could promote the immune response profile in HCC, the other three appeared to negatively regulate the immune profile of the tumor. Since the relationships between the five signature genes and macrophage M0, macrophage M1, macrophage M2, etc. in the above analysis were not intuitive, we further plotted the correlations between the signature genes and tumor immune cells based on the GEO data (Additional file 5: Fig. S2). It can be visually seen that LY6E showed a significant positive correlation with macrophage M1. The correlation between IFITM1 and macrophage M0 was negative and positive, while SPP1 showed a significant positive correlation with macrophage M0 and macrophage M2. MT1M and GADD45A did not seem to show a significant correlation with all three (*P* < 0.05).

We also analyzed the correlations between five signature genes and common immune checkpoints and showed that LY6E was positively correlated with TNFSF9, LAIR1, PDCD1, TNFRSF14, CD48, CD86, CD276, HAVCR2, TNFRSF18 and LGALS9 (r > 0.3, *P* < 0.001, Additional file 5: Fig. S3a). IFITM1 showed high positive correlations with CTLA4, CD80, CD200, CD200R1, CD274, CD44, LGALS9, HAVCR2, TMIGD2, TIGIT, CD28, ICOS, LAIR1, CD40LG, CD27, PDCD1LG2, CD86 and CD48 (r > 0.3, *P* < 0.001, Additional file 5: Fig. S3b). GADD45A was negatively correlated with TNFRSF18, TNFRSF4, CD276, and LGALS9 (r <  − 0.3, *P* < 0.001, Additional file 5: Fig. S3c). SPP1 was significantly positively correlated with CD276, HAVCR2, CD44, VTCN1, and LGALS9 were significantly positively correlated (r > 0.4, *P* < 0.001, Additional file 5: Fig. S3e). As for MT1M, it seemed to be less strongly associated with each immune checkpoint (|r|< 0.2, Additional file 5: Fig. S3d).

### Signaling pathway analysis

**Fig. 9:**
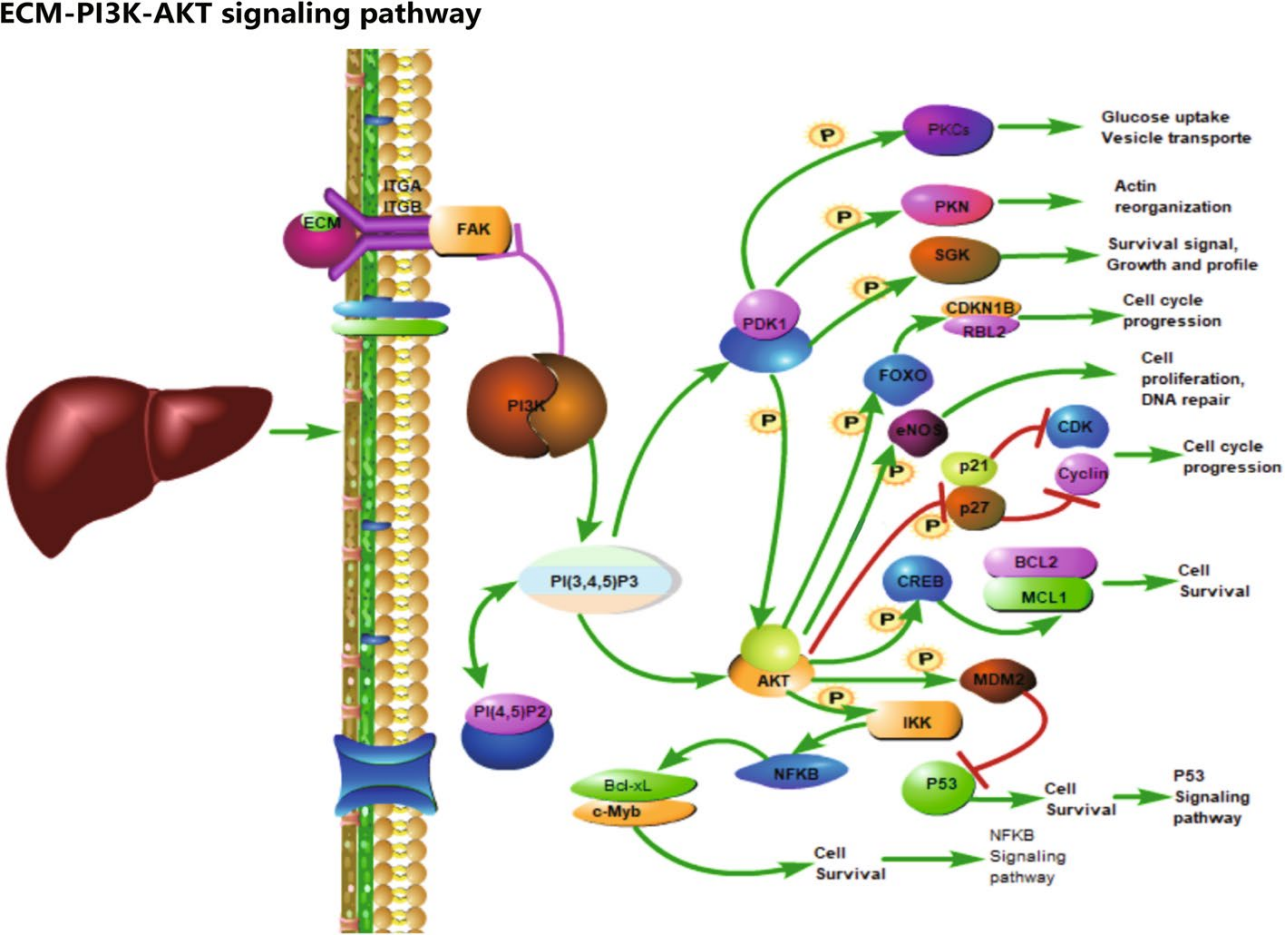
Schematic representation of the activation of PI3K/AKT signaling pathway by SPP1 through integrins

Finally, we also explored the signaling pathways that five signature genes involved, and the results showed that SPP1 is an extracellular matrix (ECM) component, which has been proven to be associated with various cancer proliferation and differentiation processes [12, 13], while ECM components and factors can affect the cell cycle by activating the PI3K-AKT signaling pathway through the integrins IGTA and IGTB, which are closely related to the proliferation and apoptosis processes of cancer cells (Fig. 9).

## Discussion

Lymphocyte antigen 6 family member E (LY6E), glycosylphosphatidylinositol (GPI) anchored cell surface protein that regulates T-lymphocytes proliferation, differentiation, and activation. Regulates the T-cell receptor (TCR) signaling by interacting with component CD3Z/CD247 at the plasma membrane, leading to CD3Z/CD247 phosphorylation modulation (By similarity). It was shown that upregulation of LY6E expression was associated with poor prognosis in several cancers [14–16], and abnormal overexpression of LY6E was found to increase the expression of the HIF-1α gene mainly at the transcriptional level in mouse models. This promotes the expression of angiogenic factors VEGFA and PDGFB, leading to an increase in tumor vascular density, and targeting this pathway for cancer therapy is effective [17]. It was also mentioned that specific antibody–drug conjugates (ADCs) of LY6E could inhibit cell proliferation in vitro and produce durable tumor regression in clinically relevant transplantation models expressing LY6E [18].

Interferon-induced transmembrane protein 1(IFITM1) is a member of the IFN-induced transmembrane protein family, which inhibits the entry of viruses to the host cell cytoplasm, permitting endocytosis, but prevents subsequent viral fusion and release of viral contents into the cytosol. And plays a key role in the antiproliferative action of IFN-gamma either by inhibiting the ERK activation or by arresting cell growth in the G1 phase in a p53-dependent manner. It has been shown that IFITM1 is tightly regulated by the proliferation, migration, and invasion of breast cancer and inhibits apoptosis, and that inhibition of IFITM1 expression by disrupting interferon-α and NF-κB crosstalk can attenuate triple-negative breast cancer progression [19, 20], which may be one of the novel immunomarkers for endometrial cancer stromal cells [20], and knockdown of IFITM1 in pancreatic cancer was also found to inhibit the proliferation of cells, that induced cell cycle arrest and apoptosis, and inhibited cancer progression [21]. In small-cell lung cancer, IFITM1 overexpression was found to promote distant metastasis, and in lung adenocarcinoma, IFITM1 expression was found to be significantly associated with increased microvessel density and correlated with patient prognosis [22]. The scarcity of IFITM1 studies in HCC so far may be related to its downregulation in HCC.

Growth arrest and DNA damage-inducible alpha (GADD45A), a member of the GADD45 gene family, is normally induced by DNA damage and other stress signals associated with growth arrest and apoptosis [23]. And the protein encoded by this gene feeds back to environmental changes through activation of the p38/JNK pathway mediated by MTK1/MEKK4 kinase. It has been shown that GADD45A is strongly associated with various cancers, for example, in pancreatic cancer, Sinapine thiocyanate (ST) can upregulate the expression level of GADD45A and exhibit significant proliferation inhibition in pancreatic cancer cells [24]. And the PI3K/AKT signaling pathway mediated by the downregulation of GADD45A is associated with reduced radiosensitivity in cervical cancer (CC) [25]. Knockdown of GINS2 in non-small cell lung cancer (NSCLC) inhibits NSCLC proliferation and promotes apoptosis through the p53/GADD45A pathway [26], while GADD45A levels were found to be lower in bladder cancer (BC) than in adjacent normal tissues, and induction of its expression inhibited the proliferation and differentiation of BC cells [27]. Silencing of GADD45A expression in cutaneous squamous cell carcinoma (SCC) was also found to increase tumor cell proliferation and reduce apoptosis and senescence through the p53 signaling pathway in cutaneous squamous cell carcinoma [28]. And it was also mentioned that GADD45A expression levels in triple-negative breast cancer seemed to exhibit good two-point performance on its risk grouping, etc. [29].

Metallothionein 1M (MT1M), is a member of the metallothionein superfamily, type 1 family. Metallothioneins have a high content of cysteine residues that bind various heavy metals. These genes are transcriptionally regulated by both heavy metals and glucocorticoids. MT1M is also highly involved in cancer, for example, in esophageal squamous cell carcinoma (ESCC) the MT1M expression levels were found to be downregulated, and after its upregulation was induced, MT1M was found to inhibit esophageal cancer cell carcinogenesis through inhibition of epithelial-mesenchymal transition and SOD1/PI3K axis [30]. While in lung adenocarcinoma MT1M overexpression was also found to inhibit A549 cell viability and migration ability, decreased MT1M expression promoted tumor cell proliferation and migration [31]. In vitro experiments showed that MT1M upregulation significantly inhibited colony formation, proliferation, migration, and invasion of thyroid cancer (PTC) cell lines, it is suggested that MT1M may be a potential new marker and target for thyroid cancer therapy [32]. In another study, it was also mentioned that MT1M was frequently downregulated in HCC, which might be associated with methylation of the promoter region, and upregulation of miR-545-3p, and lead to downregulation of MT1M, which in turn regulates the proliferation, invasion, and migration of hepatocellular carcinoma cells [33, 34].

Secreted phosphoprotein 1(SPP1), an extracellular secreted glycol phosphoprotein is closely related to the biological processes of multiple types of cancer, such as proliferation, and migration. For example, SPP1 expression in LUAD tissues was significantly higher than in normal tissues, positively correlated with TNM stage, lymph node metastasis, and depth of infiltration, and was associated with poorer prognosis by upregulating COL11A1 expression to promote cell migration and invasion [13]. And in pancreatic adenocarcinoma, SPP1 expression levels were associated with patient prognosis and immune regulation [35], and in lung adenocarcinoma, SPP1 was found to be associated with drug resistance [36]. In a recent study, researchers further investigated the level of serum SPP1 autoantibodies in patients with esophageal squamous cell carcinoma (ESCC) and showed that its expression was significantly upregulated compared to the normal group, suggesting that anti-SPP1 autoantibodies are a novel biomarker for the detection of ESCC [37], and SPP1 is important in prostate cancer (PCa), head and neck cancer (HNC), cervical squamous cell carcinoma (SCC) and other cancers [38, 39].

In present study, we found that the expression levels of LY6E, IFITM1, GADD45A, and MT1M were significantly upregulated in DM, but downregulated in HCC, and SPP1 was upregulated in both diseases. Considering that the first four genes were significantly upregulated in DM but significantly down-regulated in HCC, the two diseases showed opposite expression trends, and the expression of LY6E, IFITM1, GADD45A, and MT1M may be more efficient in identifying the occurrence of HCC in DM patients at an early stage, which provides a feasible strategy for early screening of HCC. However, more clinical trials are needed to validate this method. The expression level of SPP1 was significantly upregulated in both DM and HCC, which is a common risk factor for both diseases and plays an important role in the pathogenesis of DM and HCC. And the expression level of SPP1 can predict the onset of HCC alone or DM with HCC to a certain extent, and is an important target for predicting the development of HCC with DM. Based on the present study, we can further investigate the specific association between SPP1 expression level and clinical disease progression of patients, and thus predict the disease more accurately.

The immune profile of a tumor can be used to assess the tumor progression and treatment to a certain extent, which can be used as a reference for further treatment and disease regression. We found that the expression of all four genes was positively correlated with the infiltration abundance of tumor immune cells, except for GADD45A, which was negatively correlated with the infiltration abundance of tumor immune cells, and the expression levels of LY6E, IFITM1, and MT1M were all down-regulated in HCC, suggesting that the down-regulation of LY6E, IFITM1, and MT1M may negatively regulate tumor immune response in HCC, only GADD45A, and SPP1 may be associated with promoting tumor immune responses in HCC.

The discovery of new immune targets has been a hot topic for cancer therapy. We also investigated the correlation between five signature genes and common immune checkpoints. The positive correlation of LY6E, IFITM1, GADD45A, and SPP1 with different immune checkpoints suggested that the expression levels of checkpoints related to the four characteristic genes could be predicted to some extent by detecting LY6E, IFITM1, GADD45A, and SPP1 in HCC, and thus the sensitivity of targeted therapy. In addition, we also noted that there was a significant correlation between different immune checkpoints, and further investigation of the intrinsic association from this perspective may provide some meaningful references for the development of multi-targeted therapies.

Expression analysis showed that the expression level of SPP1 was significantly upregulated in both diseases, and we hypothesized that SPP1 expression might be a common risk factor for the development of DM and HCC, playing an important role in promoting the development of both diseases. The nomogram risk model showed that the downregulation of LY6E, IFITM1, GADD45A, and MT1M expression and upregulation of SPP1 expression in HCC are both risk factors for HCC, and contribute to the pathogenesis and progression of HCC, but the specific mechanisms are not clear and need to be investigated in a more recent study. Then, to further investigate the biological significance of SPP1 expression upregulation on HCC, we also investigated five genes related signaling pathways, and the results showed that SPP1 may activate PI3K/AKT signaling pathway through integrin ITGA and ITGB to regulate cell proliferation, differentiation, apoptosis, and other processes, which may play an important regulatory role in the occurrence and development of HCC. This may be one of the important mechanisms in the pathogenesis of HCC.

And finally, there are still many limitations. Firstly, the research data used in this research are from different public databases, so the results are affected by the source data. And in addition, the different detection methods among the research data from different sources may lead to deviations among the data, which may affect the results, too.

## Conclusion

DM signature genes LY6E, IFITM1, GADD45A, MT1M, and SPP1 are risk factors for HCC, strong association with the onset of HCC, and they show good diagnostic efficacy and are diagnostic targets for HCC. SPP1 may activate PI3K/AKT signaling pathway through integrin-mediated activation to regulate the proliferation and migration of HCC cells.

## Supplementary Information

**Additional file 1**. Original data of DM.xlsx.

**Additional file 2**. Original data of HCC.xlsx.

**Additional file 3**. DM diffGeneExp.xlsx.

**Additional file 4**. HCC diffGeneExp.xlsx

**Additional file 5: Figure S1**Venn diagram of the intersection of differentially expressed genes in DM and HCC.**Figure S2** Heat map of correlation analysis between disease signature genes and tumor immune cells based on GEO data.**Figure S3** Heat map of analysis of correlations between 5 signature genes and common immune checkpoints. And a–e in order are LY6E, IFITM1, GADD45A, SPP1 and MT1M.

## Data Availability

The data (HCC) used in this research were obtained from The Cancer Genome Atlas (TCGA, https://portal.GDC.cancer.gov/), GTEx (https://www.gtexportal.org/home/index.html) database and the GEO (https://www.ncbi.nlm.nih.gov/geo/) database (GSE46239 and GSE1551 for DM, and GSE14520 and GSE36376 for HCC), all of which are available in publicly available databases. This study complies with its data use and publication rules.
