# Supplementary material for: Risk prediction for dermatomyositis-associated hepatocellular carcinoma
Source: BMC Bioinformatics. 2023 May 31;24:222. doi: 10.1186/s12859-023-05353-6 (PMC10233868; doi:10.1186/s12859-023-05353-6)
Supplement: Supplementary file 5 — Additional file 5: Figure S1Venn diagram of the intersection of differentially expressed genes in DM and HCC.Figure S2 Heat map of correlation analysis between disease signature genes and tumor immune cells based on GEO data.Figure S3 Heat map of analysis of correlations between 5 signature genes and common immune checkpoints. And a–e in order are LY6E, IFITM1, GADD45A, SPP1 and MT1M. [file 12859_2023_5353_MOESM5_ESM.docx]

**
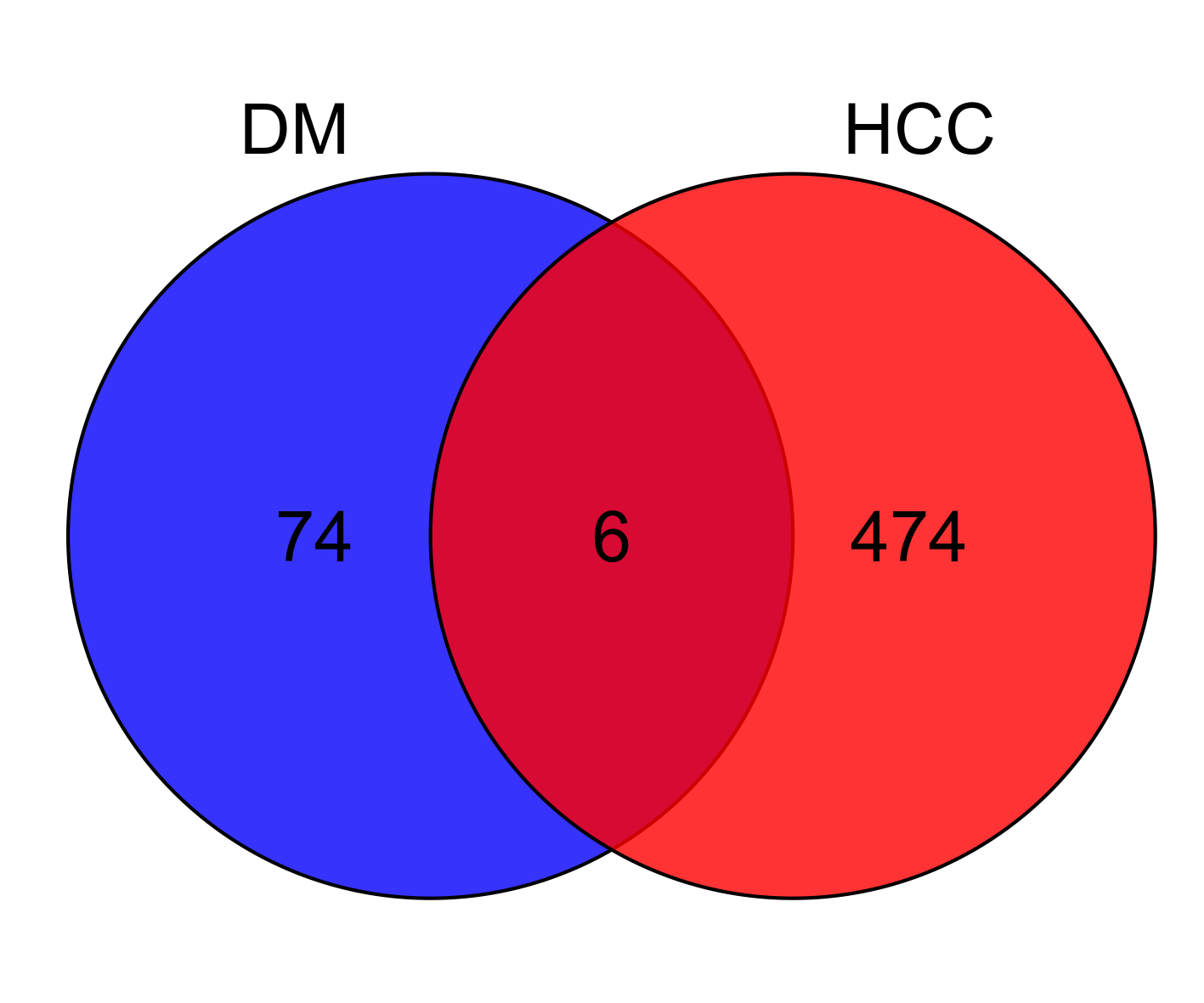
**

**Figure S1. Venn diagram of the intersection of differentially expressed genes in DM and HCC.**


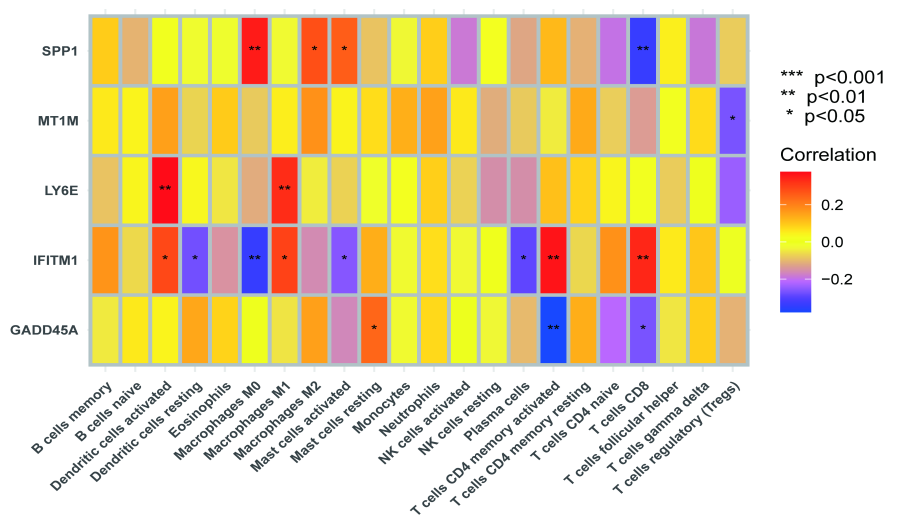


**Figure S2. Heat map of correlation analysis between disease signature genes and tumor immune cells based on GEO data.**

**
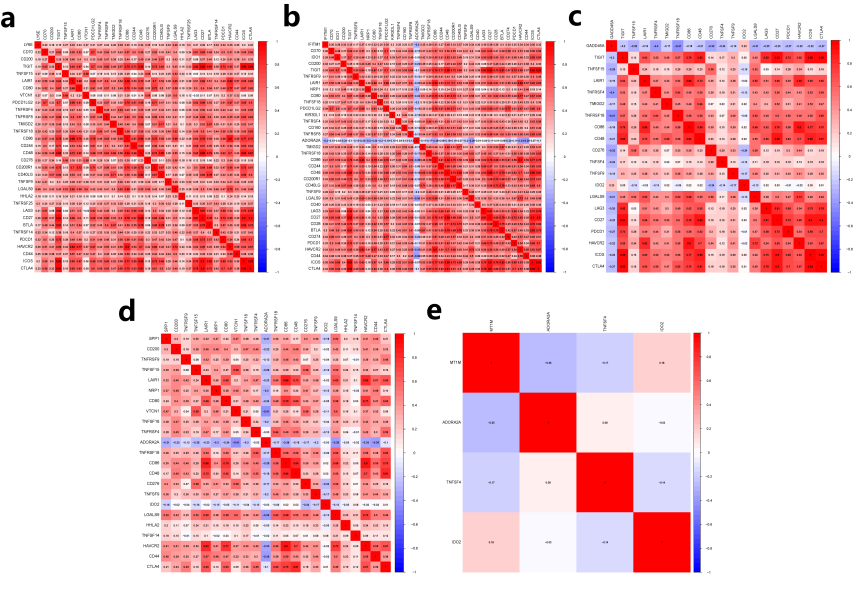
**

**Figure S3. Heat map of analysis of correlations between 5 signature genes and common immune checkpoints.** And a-e in order are LY6E, IFITM1, GADD45A, SPP1 and MT1M.
